# Supplementary material for: Does Generic Cyclic Kinase Insert Domain of Receptor Tyrosine Kinase KIT Clone Its Native Homologue?
Source: Int J Mol Sci. 2022 Oct 25;23(21):12898. doi: 10.3390/ijms232112898 (PMC9656684; doi:10.3390/ijms232112898)
Supplement: Supplementary file 1 [file ijms-23-12898-s001.zip › ijms-1956138-supplementary.pdf]

Supplementary Materials

Does generic cyclic Kinase Insert Domain of Receptor Tyrosine Kinase KIT clone its native homologue?

Julie Ledoux[1] and Luba Tchertanov[1*]

Content:

3 Figures: Figure S1; Figure S2; Figure S3

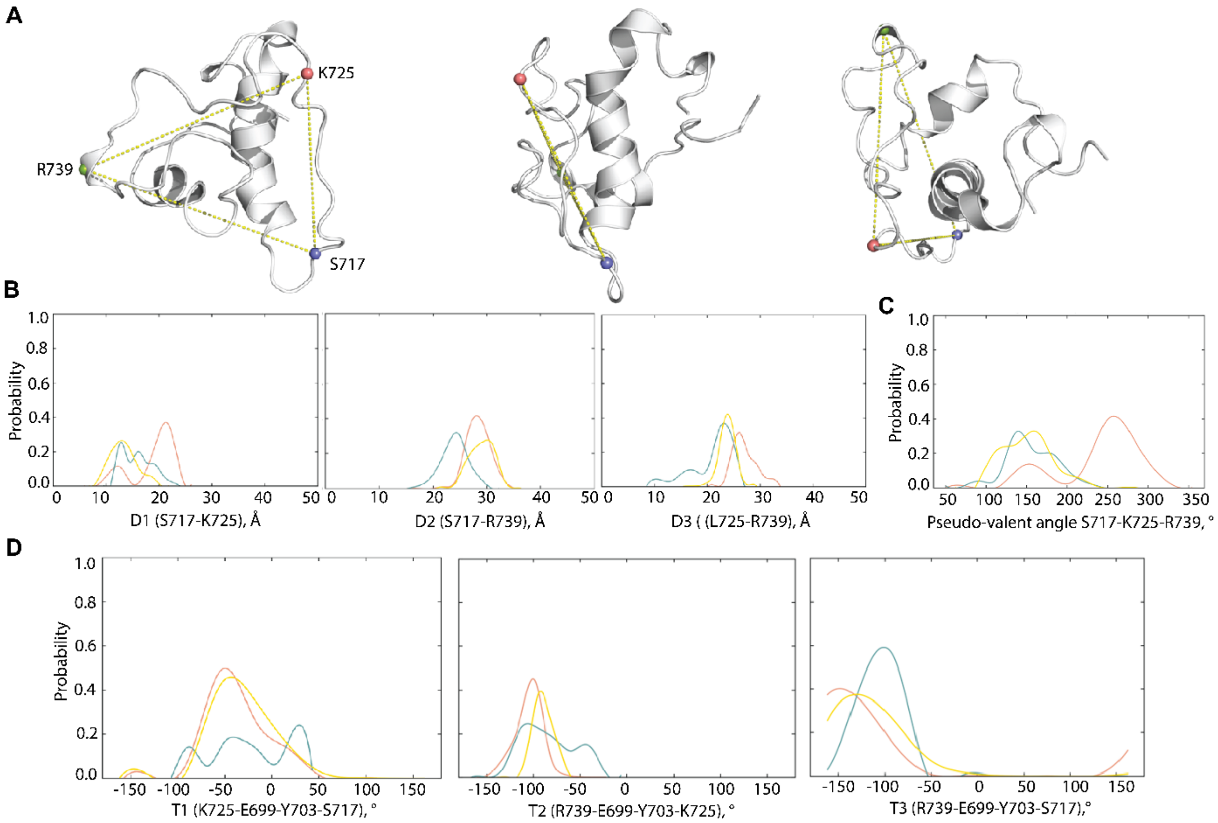

Figure S1. Geometry metrics of the most fluctuating residues. (A) 3D structure. Protein is shown as cartoon; the most fluctuating residues are displayed as balls. (B) The distance values probability. (C) The pseudo-valent angle probability. (D) Pseudo-torsion (dihedral) angle probability. (B-C) Metrics were calculated for each KID entity: KID^{GC} in yellow, KIDD in red and KIDC in teal.

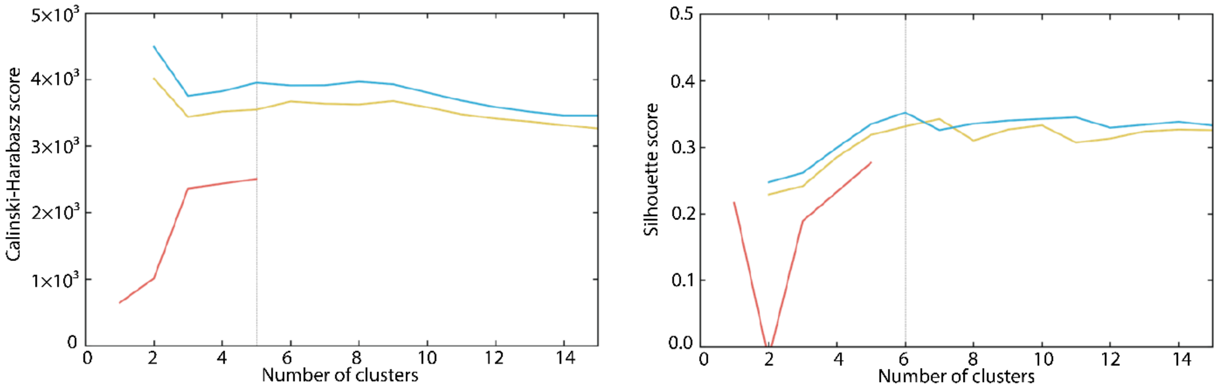

Figure S2. Performance scores were obtained during hyperparameters tweaking to find the better clustering method. Number of clusters obtained by DBSCAN [47] (in red), K-means [48] (in blue) and Ward's [49] methods (in yellow). Scores were computed with Calinski-Harabasz [51] (left) and Silhouette [50] (right).

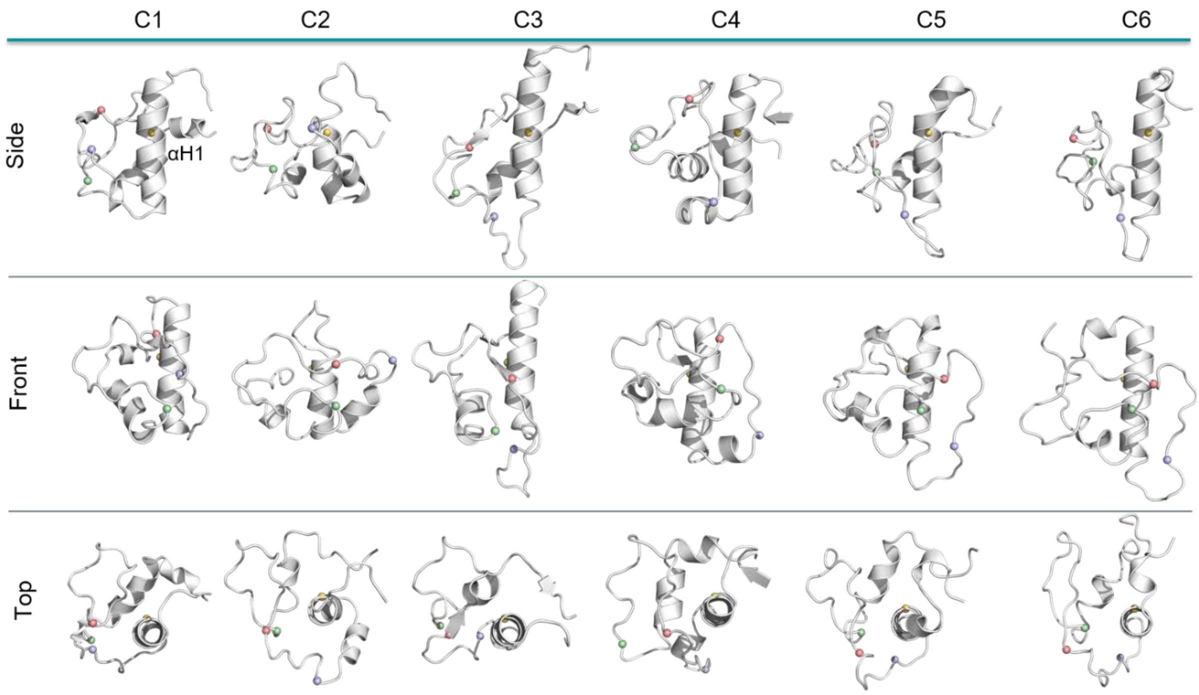

Figure S3. Representative conformations from each cluster (C1 – C6). The calculation was performed on the concatenated trajectories after fitting all data on *α*H1-helix taken from KID conformation at t=0. Three views – side, front and top – concerning *α*H1-helix are shown. Protein is displayed as cartoon, and the position of each tyrosine residue is shown by ball: Y703 in yellow, Y721 in lilac, Y730 in coral and Y747 in green.

3 Tables: Table S1; Table S2

Table S1. Non-overlapping metrics (features) value intervals in each most populated KID cluster show their differences in geometric properties.

**Cluster**		**Metric (Feature)**	**Value range**	
**C_{N1}**	**C_{N2}**		**C_{N1}**	**C_{N2}**
**C1**	**C2**	Distance S717-K725	17.1 ± 2.7 Å	12.4 ± 2.0 Å
		Distance Y721-Y747	9.6 ± 2.0 Å	14.4 ± 1.7 Å
	**C3**	Radius of gyration	12.4 ± 0.4 Å	13.7 ± 0.2 Å
		ψ Y721	-30.7 ± 34.9 °	73.9 ± 30.9 °
		ψ Y730	-18.0 ± 24.1 °	140.8 ± 29.3 °
	**C5**	Distance S717-K725	17.1 ± 2.7 Å	21.5 ± 1.7 Å
		Distance Y730-Y747	13.1 ± 3.9 Å	6.5 ± 0.8 Å

		ψ Y721	-30.7 ± 34.9 °	152.0 ± 22.0 °
**C2**	**C3**	Distance Y703-Y747	12.0 ± 1.4 Å	16.8 ± 1.2 Å
		Distance Y721-Y747	14.4 ± 1.7 Å	8.7 ± 1.0 Å
		Radius of gyration	12.4 ± 0.3 Å	13.7 ± 0.2 Å
		ψ Y721	-28.2 ± 18.6 °	73.9 ± 30.9 °
		ψ Y730	-11.6 ± 35.1 °	140.8 ± 29.3 °
	**C5**	Distance S717-K725	12.4 ± 2.0 Å	21.5 ± 1.7 Å
		Distance Y721-Y747	14.4 ± 1.7 Å	10.0 ± 1.1 Å
		ψ Y721	-28.2 ± 18.6 °	152.0 ± 22.0 °
**C3**	**C5**	Distance S717-K725	13.0 ± 2.0 Å	21.5 ± 1.7 Å
		Distance Y703-Y747	16.8 ± 1.2 Å	11.3 ± 0.8 Å
		Distance Y730-Y747	9.0 ± 1.6 Å	6.5 ± 0.8 Å
		Radius of gyration	13.7 ± 0.2 Å	12.6 ± 0.3 Å
		ψ Y721	73.9 ± 30.9 °	152.0 ± 22.0 °
		ψ Y730	140.8 ± 29.3 °	-6.9 ± 33.6 °

Table S2. Contingency table of agreement (%) on cluster population between K-means run with k = 5 and k = 6. The best population sizes are in bold.

		**k = 6**						
		C1	C2	C3	C4	C5	C6	**Total**
**k = 5**	C1	0.0	0.0	0.0	**0.2**	**21.2**	0.0	**21.4**
	C2	0.0	0.0	**22.5**	**11.9**	0.1	0.0	**34.5**
	C3	0.0	0.0	0.0	0.0	0.0	**7.1**	**7.1**
	C4	0.0	**22.9**	0.0	**0.2**	0.0	0.0	**23.1**
	C5	**13.8**	0.0	0.0	0.0	0.0	0.0	**13.8**
	**Total**	**13.8**	**22.9**	**22.5**	**12.3**	**21.3**	**7.1**	**100**
